# On Disturbed Time Continuity in Schizophrenia: An Elementary Impairment in Visual Perception?

**DOI:** 10.3389/fpsyg.2013.00281

**Published:** 2013-05-28

**Authors:** Anne Giersch, Laurence Lalanne, Mitsouko van Assche, Mark A. Elliott

**Affiliations:** ^1^INSERM U1114, Department of Psychiatry, Fédération de Médecine Translationnelle de Strasbourg (FMTS), University Hospital of StrasbourgStrasbourg, France; ^2^Department of Psychology, National University of Ireland GalwayGalway, Ireland

**Keywords:** schizophrenia, time, anticipation, synchrony, attention, implicit processing, Simon effect

## Abstract

Schizophrenia is associated with a series of visual perception impairments, which might impact on the patients’ every day life and be related to clinical symptoms. However, the heterogeneity of the visual disorders make it a challenge to understand both the mechanisms and the consequences of these impairments, i.e., the way patients experience the outer world. Based on earlier psychiatry literature, we argue that issues regarding time might shed a new light on the disorders observed in patients with schizophrenia. We will briefly review the mechanisms involved in the sense of time continuity and clinical evidence that they are impaired in patients with schizophrenia. We will then summarize a recent experimental approach regarding the coding of time-event structure in time, namely the ability to discriminate between simultaneous and asynchronous events. The use of an original method of analysis allowed us to distinguish between explicit and implicit judgments of synchrony. We showed that for SOAs below 20 ms neither patients nor controls fuse events in time. On the contrary subjects distinguish events at an implicit level even when judging them as synchronous. In addition, the implicit responses of patients and controls differ qualitatively. It is as if controls always put more weight on the last occurred event, whereas patients have a difficulty to follow events in time at an implicit level. In patients, there is a clear dissociation between results at short and large asynchronies, that suggest selective mechanisms for the implicit coding of time-event structure. These results might explain the disruption of the sense of time continuity in patients. We argue that this line of research might also help us to better understand the mechanisms of the visual impairments in patients and how they see their environment.

## Introduction

Patients with schizophrenia are known to suffer from cognitive disturbances that affect their every day life and might subtend their clinical symptoms. Abnormal visual perception represents one such impairment. The deficits are diverse, affecting visual organization in space, the processing of low-spatial frequencies, and the pattern of eye movements. Here we offer an attempt to characterize abnormal visual perception in schizophrenia from a different, non-exclusive perspective, i.e., a time perspective. All aspects of mental life involve coding the succession of events in time with disturbance in the processing of events structure of likely influence on any one or more of a number of cognitive functions, in this case the cognitive functions leading to the perception of visual structure. The hypothesis of an elementary time disorder playing a central role in psychosis has been developed earlier by psychiatrists, including Minkowski (1933) as well as Andreasen (1999) who proposed the concept of a cognitive dysmetria. The types of cognitive impairment covered by cognitive dysmetria still remain to be defined. However, in essence those impairments are temporal and here we focus on the effects of a basic disturbance in temporal coding. We will review current evidence for an elementary impairment at coding the temporal events structure in patients with schizophrenia, and consider its possible impact on the general perception of the world from the point of view of the patients. We are especially interested in understanding the mechanisms subtending the sense of time continuity and its disturbance in patients with schizophrenia. Healthy subjects feel that time is flowing without interruption; they know that what has just happened is past, and expect something to happen next. This ability to follow events in time, and the expectation of the moments to come would provide its dynamism to psychic life and would be at the root of what has been called the “vital dynamism” by phenomenologists (Minkowski, 1933). In contrast, several psychiatrists have suggested that the sense of time continuity is disturbed in patients with schizophrenia. Patients would experience a fragmentation of the normal flow of events (Fuchs, 2007; Vogeley and Kupke, 2007) and a loss of “vital dynamism” (Minkowski, 1933). Approaching the phenomenological experience of time in patients with schizophrenia is complex, particularly given patients’ typical difficulties in reporting their inner mental life. Patients nonetheless report disturbances that can be taken as indications as to how they experience their disorder. Interestingly, the patients’ descriptions often relate to their experience of visual events. For example, Chapman (1966) reports several patients’ descriptions, including the following:

Things go too quick for my mind […]. It’s as if you were seeing one picture 1 min and another picture the next.

When I start walking I get a fast series of pictures in front of me. Everything seems to change and revolves around me. Something goes wrong with my eyes and I’ve got to stop and to stand still.

It is difficult to derive from these descriptions which cognitive mechanisms are really disturbed in patients, but many disturbances related to time have also been described at an experimental level in patients with schizophrenia, including in particular the disturbed perception of event duration (Volz et al., 2001; Elvevåg et al., 2004; Davalos et al., 2005; Allman and Meck, 2012). The studies are based on the theory that there is an internal clock allowing us to count time and the results are interpreted in terms of a possible change in the rhythm of the clock (although see Delevoye-Turrell et al., 2012; Roy et al., 2012; Turgeon et al., 2012). However, it is unclear how a change in the internal clock relates to a disturbed sense of time continuity. Hence to explore the latter, we considered a different theoretical framework aimed at understanding time-event structure and time continuity.

Mechanisms involved in the sense of time continuity may operate at various time scales (reviewed in Wittmann, 2011). The experimental evidence points toward the existence of elementary time windows providing us a lower margin in our ability to distinguish separate events in time. Even though two spatially separate stimuli are presented at different times for very brief intervals they may appear to be presented simultaneously. Exner (1875) suggested minimum time differences in temporal order discrimination for intervals of up to 17 and 44 ms. Considering invariances, a common measure that extends across sensory modalities seems to be the minimum time required for temporal order discrimination following the successive presentation of more than two stimuli. For tactile and visual stimuli, and irrespective to the precise structure of the visual stimuli concerned, simultaneity thresholds have been determined with remarkably little variation: Brecher (1932) showed what he referred to as units of “subjective time” corresponded to average periods of 55.3 ms for tactile stimulation and periods of 56.9 ms for visual stimulation, with standard deviations of no greater than 1.4 ms.

Brecher’s as well as subsequent and related empirical demonstrations of simultaneity thresholds in the mid 50 ms region have been interpreted in terms of a window of time, or a perceptual moment within which all events will be processed together leading to them being perceived as occurring simultaneously (von Baer, 1864; Lalanne, 1876; Brecher, 1932; Elliott et al., 2006, 2007; van Wassenhove, 2009; Wittmann, 2011). Although referred to in terms of perception, time windows of this order of magnitude would represent an elementary quantum that does not necessarily relate directly with experienced duration. Only larger time windows of up to 2–3 s would be associated with the experience of duration and these would represent a second or subsequent interval scale related to the sense of time continuity. Across these longer intervals information would not be temporally fused and perceived in terms of a perceptual unity but would be separate events grouped together within the same moment of experience. A popular example is the experience of present time arising when listening to a melody: when listening to the present tone, the previous tone is still in mind, while the coming tone is usually anticipated. Because past, present, and future tones are all momentarily present in mind, all of them are part of the subjective present. The past tone is nonetheless known as being past, and is thus both past (in objective time) and present (in subjective present time). Similar reasoning holds for the future tone, and this leads to the concept of specious present (James, 1896). Husserl (1893/1917) has proposed that the integration of past, present, and future represents a key mechanism in our sense of time continuity. It is not known to which amount and how the shorter 30 ms time windows are integrated within the experienced 3 s moments (Pöppel, 1997, 2009; Szelag et al., 2004). This requires an understanding of how windows overlap and integrate with one another (Elliott, 2005; Dainton, 2010). It might be proposed that temporal windows and their overlap are brought about by neuronal synchronization (Varela, 1999). Because neuronal synchronization of action potentials requires time, even the processing of the simplest event, such as the display of a square on a computer screen, is time-consuming and so coded within a temporal window of some duration. Different events would then overlap in time even if their onset is shifted in time. The result would be a sense of continuity rather than the perception of discrete moments.

There are thus several candidate mechanisms bringing about a sense of time continuity: the ability to relate past, present, and future moment, the hierarchical organization of elementary and longer time windows, or the overlap between successive elementary time windows. This means several possibilities to explain the disruption of the sense of time continuity in patients with schizophrenia. However, what we currently know about time continuity might not be enough to understand the mechanisms of its disruption and a number of questions first require clarification: we might ask whether events judged to be simultaneous are really processed as co-temporal? Further, are there other means that can bring about the sense of time continuity? Finally, at which time scale does the sense of time continuity emerge? These questions require answers if we want to understand why patients with schizophrenia suffer from a disrupted sense of time continuity.

We have chosen to focus on very short time scales below 100 ms, with the aim of assessing the elementary mechanisms that subtend processing of successive stimulus events in patients. A focus on short time scales was motivated by several results from the literature: elementary timing mechanisms have been related directly with neuronal synchronization (reviews in Elliott, 2005; Elliott et al., 2006; van Wassenhove, 2009) which seems appropriate given that schizophrenia is very precisely characterized by abnormalities in synchronization at frequencies in the EEG gamma band (i.e., at around 40 Hz), and thus correspond to intervals of around 25 ms (reviewed in Uhlhaas and Singer, 2010). In addition, impairments are also observed at a behavioral level that may be explained by elementary time disorders, such as in motor actions (Delevoye-Turrell et al., 2003; Carroll et al., 2009): for example, when participants lift an object or hit a target with an object, the peak grip force is usually synchronized with the time of impact or maximal load. In patients however, grip force is delayed by around 30–100 ms (Delevoye-Turrell et al., 2003). This means a difficulty at precisely synchronizing grip force in time. Another example of abnormalities at short time scales can be found in visual perception tasks: it has been proposed that patients have difficulties to efficiently allocate attention in time, i.e., to focus attention at precise moments in time, when two stimuli follow each other at delays of between 50 and 250 ms Stimulus-Onset Asynchrony (SOA) (Granholm et al., 2009; Lalanne et al., 2012a).

We first explored the length of the elementary time window in patients with schizophrenia and found elementary time windows to be altered in patients (Giersch et al., 2009). However these disturbances led us to wonder about the processing of events within the time windows themselves. To address such questions, we devised a new method of analyzing participants’ responses. The results question the usual assumption that events are treated as co-temporal within temporal windows, and lead us to reconsider the mechanisms underlying the sense of time continuity and to propose an explanation for its disruption in patients with schizophrenia. We summarize our findings in the following^1^.

## Measuring the Windows of Visual Simultaneity

Visual stimuli separated in time by an SOA of less than 20–30 ms are judged as being simultaneous. Our aim was to compare the length of the time window in patients with schizophrenia against that found in healthy participants. The paradigm used to determine this window is relatively simple: two visual stimuli (two bars or two squares, for instance) are shown on a computer screen. They appear either simultaneously or with a short asynchrony and participants decide whether the two stimuli are simultaneous or asynchronous. In our experiments, participants responded by pressing a left response key in case of simultaneity or a right response key in case of an asynchrony. To date, four studies have shown that patients require larger asynchronies than healthy participants to report two stimuli as appearing at different times (Foucher et al., 2007; Giersch et al., 2009; Schmidt et al., 2011; Lalanne et al., 2012b). Several possible confounds have been eliminated, i.e., the role of eye movements, interhemispheric transfer, modality specificity, bias, and access to consciousness. Hereafter, we review shortly how we can rule out these possibilities.

It has been shown that the impairment persists if patients are required to look at a central fixation point until the stimuli appear (Lalanne et al., 2012b) indicating that the impairment is not related to abnormal eye movements (Phillips and Davis, 1994; Holzman, 2000; Trillenberg et al., 2004). In addition, impairments are similar when stimuli are displayed within the same hemifield as across hemifields (Lalanne et al., 2012b), thus eliminating the influence of impaired interhemispheric connections (Schwartz et al., 1984; Mohr et al., 2008; Knöchel et al., 2012; but see David, 1993). A difficulty to discriminate simultaneous from asynchronous events is also observed in the auditory (Foucher et al., 2007) as well as in cross-modal conditions (i.e., audio-visual, Martin et al., 2013) and finally, a possible decisional bias has been carefully ruled out by both Giersch et al. (2009) as well as Schmidt et al. (2011). A decisional bias is independent of perceptual ability and may occur during response selection: in this case it might be that patients process asynchronies as do the healthy participants but need larger asynchronies to select an “asynchronous” response. Classical methods based upon signal detection theory (Green and Swets, 1974) showed no difference between the bias of patients and healthy participants (Giersch et al., 2009; Schmidt et al., 2011).

**Figure 1:**
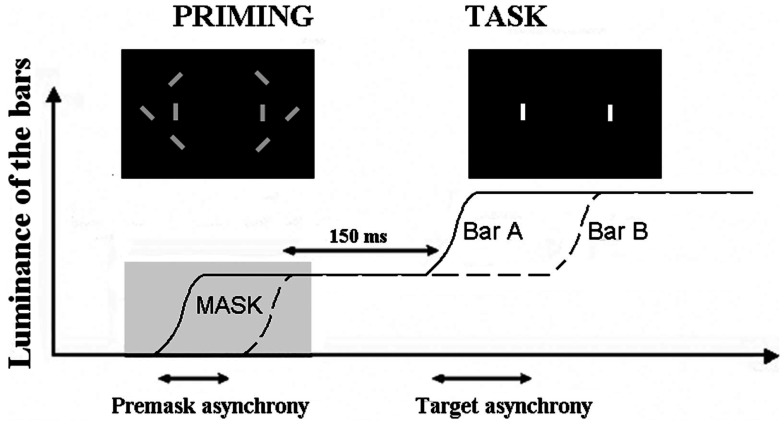
**Illustration of the paradigm used to check for a non-specific effect of subjective judgments**. The curves represent the increase in luminance of the two target bars, A and B. The first increase in luminance is used as a prime and masked by the distracters (“priming” figure). The prime is asynchronous when the two bars increase their luminance asynchronously. The task of the participant is to decide whether the second increase in luminance is simultaneous or asynchronous (with the permission of Schizophrenia Bulletin).

We used an additional method, based upon priming, which bypasses the problem of an explicit judgment on the part of the patients. The possibility of impairment due to the explicit nature of the response is distinct from a decisional bias. A deficit at making an explicit judgment is related to the mechanisms allowing information to become available to consciousness (Del Cul et al., 2006), whereas decisional biases are related to variations in the use of information to give a response. Each time a patient is explicitly asked to make a judgment, and shows abnormal performance, it can be asked whether the impairment is related to the difficulty the patient experiences in formulating his/her judgment. Patients have often been shown to be thus impaired although implicit information processing remains unimpaired (Del Cul et al., 2006). In the present context an apparent difficulty to detect an asynchrony between two items might be due to a non-specific difficulty at making a subjective judgment rather than impairment in processing the timing of events. In our task, we used a procedure developed by Elliott et al. (2007) and took an implicit measure of the effects of an asynchrony on a subsequent explicit judgment of simultaneity/asynchrony. In this paradigm two priming bars were displayed on a computer screen, either simultaneously or asynchronously while a series of six distracter bars were rapidly switched on and off around the priming bars, thus making the temporal relation between the priming bars impossible to accurately report. After the distracters switched off the priming bars remained on screen and after a short interval increased in luminance separately and with a variable SOA (which included a simultaneous increase) (Figure 1). Participants reported whether this luminance increase was simultaneous or asynchronous across the two bars.

**Figure 2:**
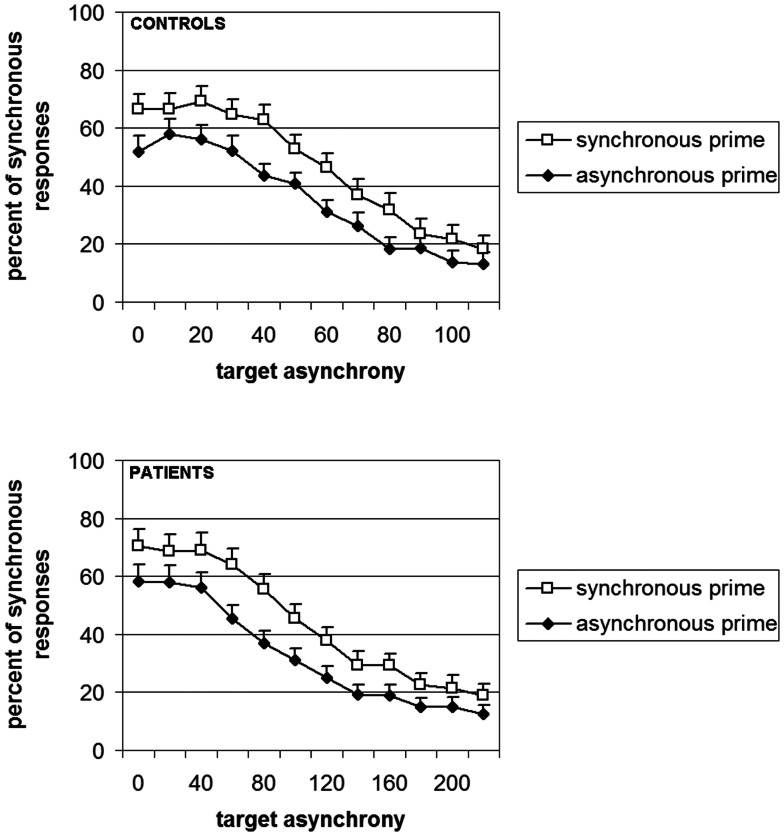
**Percent of responses “simultaneous” as a function of the prime (white squares = synchronous vs. black diamonds = asynchronous) and the group (controls in the upper panel and patients in lower panel)**. It is to be noted that the abscissa is the double for patients as compared to controls, thus illustrating the difficulty of the patients to emit a judgment in this experiment (with the permission of Schizophrenia Bulletin).

Elliott et al. (2007) showed that in healthy volunteers, the masked asynchronous bars biased participants toward reporting an asynchrony in the subsequent increase in bar luminance. We showed, in addition, that this bias increases with the asynchrony between priming bars: the larger this asynchrony, the larger the bias (Giersch et al., 2009). This was observed even though the asynchrony in the prime was individually adapted so that it was below threshold in all cases, indicating that the effect is due to the implicit priming of the asynchrony. We reasoned that if the difficulties of patients are due to impaired explicit judgments *per se*, there should be dissociation between implicit and explicit processing of the asynchrony: explicit judgments of asynchrony would be impaired while their implicit influence would remain unaffected. If this were the case, we expected priming to be identical between groups for equivalent sub-threshold asynchronies. In our case however, sub-threshold asynchronies were derived from explicit judgments and adapted individually. As a consequence they were not equivalent across groups, i.e., the asynchronies used to test the priming effect were larger in patients. Since the priming effect increases with the amplitude of the sub-threshold asynchronies, difficulty restricted to explicit judgments should have resulted in larger priming effects in patients with schizophrenia. But this was not what was observed. Instead, near identical priming effects were found for each group (Figure 2). This indicates that the enlarged temporal window observed in patients presents a true difficulty at discriminating events over time that is not explicable in terms of a non-specific difficulty in formulating the required judgment. It is to be noted that these results did not allow us to compare the implicit processing of asynchronies in patients and controls, since sub-thresholds asynchrony were not equivalent across groups. The results mainly suggest that patients require larger asynchronies than controls to reach the same perceptual level (i.e., a level that yields a priming effect), independent of the need to give an explicit response.

Inasmuch as the subjective experience of the present is built upon elementary time windows, it can be expected that a lengthened interval of subjective simultaneity distorts the sense of time continuity. Before drawing this conclusion however, we questioned whether this lengthened interval entailed the temporo-perceptual fusion of all the events within the associated temporal window, i.e., whether it meant that events are treated as co-temporal even at an implicit level. It was indeed surprising to observe the amplitude of the deficit in some patients with schizophrenia, who were unable to detect an asynchrony of more than 100 ms. Did this really mean that patients fuse events in time over periods longer than 100 ms? The next step was thus to check the integrity of implicit information processing within the window of simultaneity.

## Implicit Processing of Information within the Temporal Window

**Figure 3:**
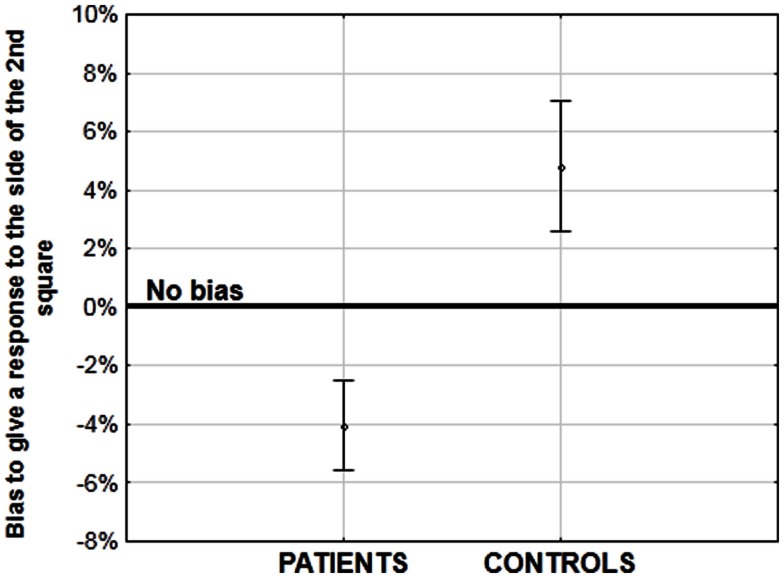
**Amplitude of the bias (in%) to the side of the first or second stimulus at short asynchronies, when one stimulus is displayed on the right side of the screen and the other one on the left**. A negative bias corresponds to a bias to the side of the first stimulus (in patients), whereas a positive bias corresponds to a bias to the side of the second stimulus (in healthy participants). See Lalanne et al. (2012b), for more detailed results.

In order to evaluate the quality of implicit information processing within the 55 ms integration window, we devised an original method of analysis, by examining the Simon effect. The Simon effect refers to the speeding of and more accurate responses when a visual stimulus is presented within the same perceptual hemifield as the responding hand (Hommel, 2011a). The precise mechanisms of the effect are a matter of discussion (i.e., Hommel, 2011a,b; van der Lubbe and Abrahamse, 2011), but the important point is that the effect is independent of the explicit instructions given to the participant. In our paradigm, it allowed us to check whether or not the stimuli are fused in time when the asynchrony is not consciously detected. During our task, two stimuli are displayed on the screen, one to the left and one to the right and participants give their response – “simultaneity” or “asynchrony” by pressing the left or right response key, respectively. When the two stimuli are displayed simultaneously, a Simon effect cannot occur: the participants cannot be biased to respond on any particular side since the displayed information is equivalent to both sides. However, when the stimuli are asynchronous there is an asymmetry and under these conditions we observed a Simon effect (Lalanne et al., 2012b,c). Healthy participants were systematically biased to answer to the side of the second stimulus independent of its right or left location and independent of the asynchrony amplitude (Lalanne et al., 2012c). Given that physical information is identical on both sides, it is only the temporal difference between the two stimuli that can lead to such a Simon effect. The important point is that this Simon effect was observed even at short asynchronies when participants reported the majority of stimulus presentations to be simultaneous (Lalanne et al., 2012b,c). This suggests that the asynchrony is processed even though participants are unable to report it. Interestingly, a Simon effect was also observed in patients with schizophrenia. At large asynchronies patients were biased to the side of the second stimuli to the same extent as were the healthy participants (Lalanne et al., 2012b). However at small asynchronies, patients were biased to the side of the first stimulus although the controls still showed a bias toward the second stimulus (Figure 3; Lalanne et al., 2012b,c). This effect has been observed in three different studies, with three different groups of 18–20 patients with schizophrenia. It has been observed for asynchronies of 8–17 ms, i.e., at delays that lead to rates of “simultaneous” responses identical to the rates observed for perfect synchrony (Lalanne et al., 2012c). As with healthy participants, this means that the asynchronies are processed in patients with schizophrenia even for delays of below 20 ms and are not fused in time. Had the stimuli been fused in time, then the two stimuli would have been processed as if identical. In this case, the Simon effect could not have occurred, i.e., the subjects could not have been biased to either stimulus. These results show that patients process very short asynchronies similarly to (but not quite in the same way as) healthy participants even though they need much larger asynchronies to explicitly report them. The Simon effect observed at asynchronies below 20 ms can be considered as implicit not only because the effect is independent of the instructions, but also because asynchronies below 20 ms are not detected and cannot drive a response bias. This contrasts with the Simon effect observed at larger asynchronies: the perception of a temporal order between two stimuli that are clearly asynchronous might drive subjects to switch attention toward the last occurring event. This cannot occur at short asynchronies. All in all this means that the Simon effect differentiates patients from controls only at short asynchronies, when temporal processing is implicit. These data show a clear dissociation between the implicit response – indicated by the Simon effect occurring at 8 and 17 ms asynchronies, and the subjective experience of temporal relations between stimuli occurring at larger asynchronies – indicated by the explicit judgments of those relations. A similar dissociation has also been observed with multisensory stimuli (Martin et al., 2013). Our results do not indicate a preserved implicit processing however: on the contrary, the results in patients with schizophrenia differ qualitatively from those in healthy participants: at short asynchronies, the responses of healthy participants are biased to the side of the second stimulus whereas the responses of patients are biased to the side of the first. Hence and consistent with our previous studies (Giersch et al., 2009) it seems that both the implicit and explicit processing of asynchronies is affected in patients with schizophrenia: the implicit impairment is revealed by the Simon effect at short asynchronies, and the explicit impairment reflects in the difficulty to explicitly report an asynchrony.

The critical question at this stage is the meaning of the bias to the side of the first or second stimulus. Bias to the side of the second stimulus might be related with studies showing that temporal coding is more precise for events offsets than onsets (Bair et al., 2002; Clifford, 2002; Tadin et al., 2010). Although there is no offset in our studies since stimuli stay on the screen until subjects give their response, a stimulus offset and the second stimulus in our study both represent the end of an event. This bias toward an event’s end initially seems surprising given the effect of prior entry. The latter has been demonstrated using similar tasks involving simultaneity/asynchrony or temporal order judgments on successive or simultaneous stimuli (review in Spence and Parise, 2010): it has been frequently shown that cueing to the first stimulus in a sequence of two facilitates subsequent judgments of order or of simultaneity/asynchrony. A cue, for example an indicator or a flash encourages deployment of attention to the cued location. This is believed to expedite processing of the first stimulus and thus facilitates detection of an asynchrony, or of a succession between first and second stimuli (Spence and Parise, 2010). The prior effect thus shows that focusing attention on the first stimulus in a sequence is important for the processing of sequential order. By contrast, we found that healthy participants were biased to the second stimulus. The major differences between the two observations is, first, that our observations take place when subjects are not aware of any asynchrony, and second, that prior entry takes place before the critical stimuli are presented. In contrast the Simon effect is recorded at the time of the participant’s response, i.e., after all stimuli are presented. From these observations it is tempting to conclude that healthy participants proceed from processing the first to the second stimulus during the sequence as if biased toward the second. Conversely, patients remain fixed on the first stimulus.

**Figure 4:**
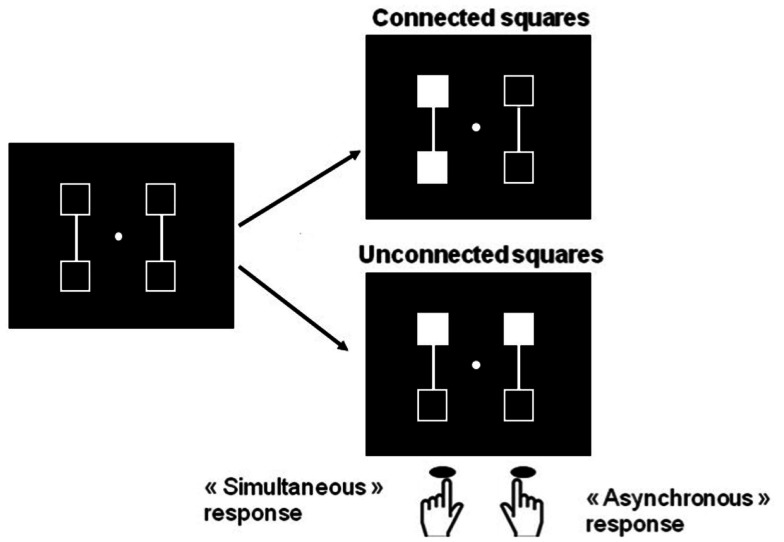
**Illustration of the procedure designed to compare simultaneity/asynchrony discrimination for connected and unconnected squares, and squares displayed in the same hemifield vs. in different hemifields**. Two squares are filled in, in gray, either simultaneously or asynchronously. These two squares are either connected or not. Participants are instructed to respond with the right key when they think the squares are filled in asynchronously and the left key when they think filling-in occurs simultaneously. In the examples, filled squares are connected and located within the same hemifield in the right top figure, and unconnected and in different hemifields in the right lower figures. The intra- vs. inter-hemifield manipulation has been conducted without connecters in a first experiment (Lalanne et al., 2012b), and with connecters in a second, distinct experiment (Lalanne et al., 2012c). The examples correspond to the second experiment. In this experiment, the location of the connecters (vertical vs. horizontal), and the location of the targets (within the same vs. in different hemifields) yielded four main possibilities (targets connected within the same or different hemifields, and targets unconnected within the same or different hemifields), which were equally represented and displayed in random order.

A number of difficulties and questions still remain with this interpretation: first it might be possible that deployment of visuo-spatial attentional mechanisms rather than any disorder in temporal processing accounts for the difficulties in task performance presented by patients with schizophrenia. Given that the two stimuli differ not only on their time onset but also on their spatial location, it might be asked whether patients have a difficulty to shift attention from the first to the second stimulus as a result of spatial or visual organization impairments in attentional mechanisms. In fact and related to this, patients with schizophrenia are known to be impaired at grouping items when those items are spatially separate (review in Silverstein and Keane, 2011) and this may make it difficult to sequentially process successively presented stimuli. To check for this possibility, we used the fact that patients’ difficulties with perceptual grouping are alleviated when clear grouping cues relate the stimuli (Giersch and Rhein, 2008; van Assche and Giersch, 2011). We adapted our paradigm and added line-segments between the stimuli in order to facilitate grouping (Figure 4).

**Figure 5:**
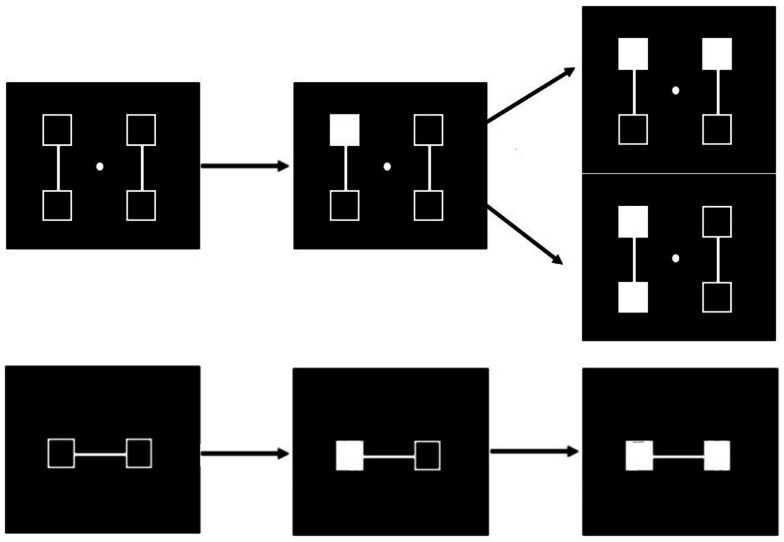
**Illustration of the successive events in the simultaneity/asynchrony discrimination task in case of an asynchrony (from left to right)**. When four locations are used (upper row) and the first square is filled in (middle figure in the upper row), there are two possible locations for the second one (figure on the right), and there is thus an uncertainty regarding the location of the second stimulus. The spatial location of the second stimulus is always predictable when only two locations are used (lower row). These two experiments have been conducted in two different groups of participants (Lalanne et al., 2012c).

We also manipulated the spatial predictability of the second stimulus to further facilitate a shift in processing toward this stimulus in patients: in one experiment there was only two possible locations for the stimuli and the second stimulus location was always predictable, whereas in a second experiment, four locations were used, and the location of the second stimulus was uncertain (Figure 5).

**Figure 6:**
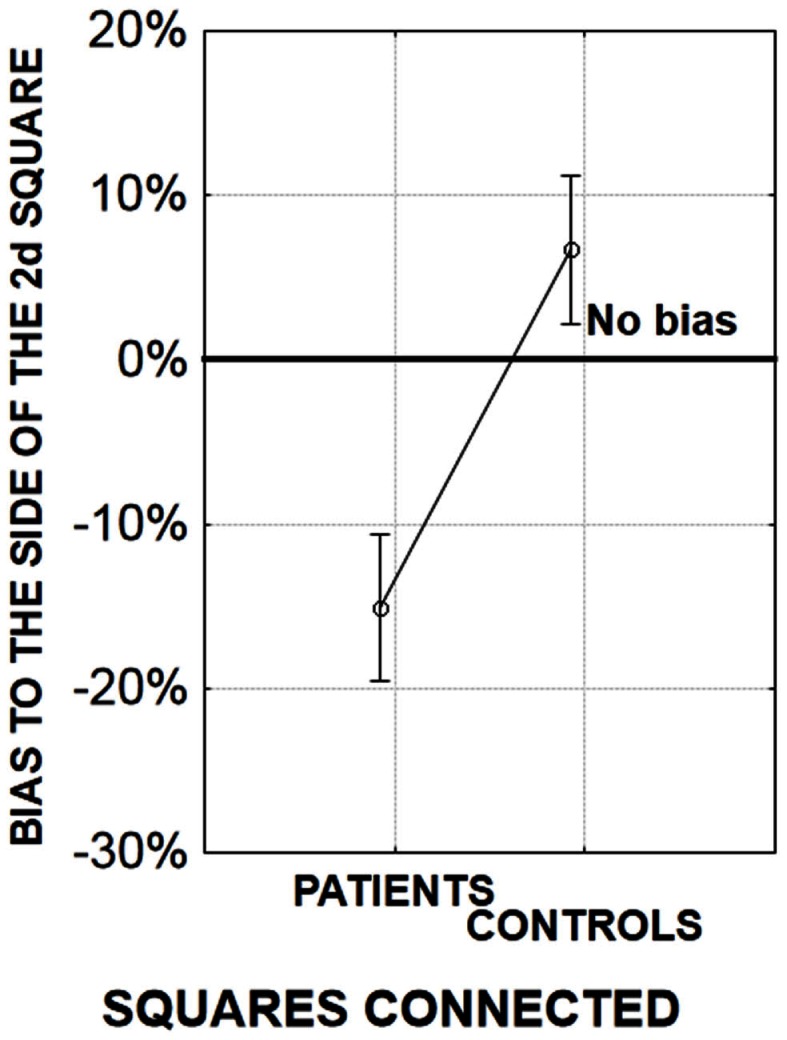
**Amplitude of the bias (in %) to the side of the first or second stimulus for an asynchrony of 17 ms**. A negative bias corresponds to a bias to the side of the first stimulus (in patients), whereas a positive bias corresponds to a bias to the side of the second stimulus (in healthy participants). In the present experiment, there were two possible locations for the squares, one on the left part and one on the right part of the screen. As a consequence, the location of the second square was always predictable (similar results were observed when the location of the second square was more uncertain, Lalanne et al., 2012c). The squares were either connected (the displayed graph) or unconnected (data not shown, but see Lalanne et al., 2012c).

Since perceptual grouping promotes the perception of simultaneity (Nicol and Shore, 2007), it was expected that all Simon effects would be reduced by making right and left stimuli more similar in their temporal properties. As expected, the bias shown by healthy participants’ to the side of the second stimulus was reduced when stimuli were connected (Lalanne et al., 2012c). On the other hand, the Simon effect to the side of the second stimulus was increased when the location of the second stimulus was predictable. The results in healthy participants thus confirmed that our experimental manipulations had the expected effect. However in patients there was a very clear bias to the side of the first stimulus when the first and second stimulus were related with a line-segment, and when the second stimulus’ location was predictable in 100% of the cases (Figure 6).

This suggests that in patients the abnormal bias to the side of the first stimulus persists when grouping and spatial difficulties are alleviated by experimental manipulations. Overall and in summary, these results suggest difficulties related to time rather than to spatial and visual organization impairment.

It should be noted that it is unlikely that the implicit processing of asynchronies involves a coding of succession. This point is critical when considering the sense of time continuity. As emphasized above, it has been proposed that the sense of time continuity arises from the integration of past, present, and future moments within the subjective present (Husserl, 1893/1917). Succession is a way of establishing a link between past, present and future events, and it would represent a mechanism of integration. If this occurs within elementary time windows, it would mean that integration of past, present and future takes place on a shorter time scale than previously believed. Several empirically founded arguments speak against this possibility: first, the physical characteristics of the stimuli, i.e., their spatial and temporal separation make it unlikely that the Simon effect is mediated by a coding of motion between the two successive stimuli (see Lalanne et al., 2012c, for a complete discussion on this point). Besides, it would be surprising that events are coded one relative to another on time scales shorter than 20 ms. Automatically ordering events in time might indeed be costly. In our experiments stimuli were sometimes displayed in two different hemifields and thus processed in different cerebral hemispheres (e.g., Figure 2). Comparing their onsets is bound to involve long-distance connectivity (at least to transfer information regarding the stimuli onset) and some specialized comparison mechanisms. If this is to be generalized in the natural environment, it would mean permanent comparisons between unrelated stimuli. The usefulness of such computation might be questioned. In addition the computation cost would increase exponentially in a crowded environment. It might thus be proposed that even if events delayed by short asynchronies are not processed as being co-temporal, their succession is not coded automatically. This possibility is supported by our most recent results in healthy participants (Giersch et al., in press).

Even if the lack of succession coding makes it impossible to tag stimuli as “before” and “after,” the second stimulus might still be identified as the last occurring event. In fact, a bias to the side of the second stimulus in a succession of two suggests the existence of a mechanism assigning priority to the last occurring event. This might be analogous to what happens when attention is driven reflexively by an external event. It is known that attention deploys to novel information, meaning that associated brain mechanisms are designed to continually check for novel information, not only in space but also in time. Feedback loops described in visual perception, predictive coding, or forward models might provide neural bases for these mechanisms (Miall et al., 1993; Wolpert et al., 1998; Elliott and Müller, 2000; Kompass and Elliott, 2001; Friston, 2008). Although the results suggest some kind of prioritization and possibly an involvement of attention, the processes making this prioritization possible are not available to consciousness. Participants do not perceive very short asynchronies, and they put nonetheless more weight on the second and last stimulus. Even if this entails the involvement of attention, it cannot be induced by conscious expectation.

What is the relationship between the ability to distinguish stimuli at very short asynchronies and the sense of time continuity? In so far as succession is coded only at longer delays, the integration of past, present and future moments, proposed as a mechanism of “time continuity” defines only delays of that order of magnitude. However, what is observed at the shortest delays is an automatic priority for the latest events, possibly sub-tended by mechanisms allowing us to permanently look ahead or anticipate future events. It might be asked whether this bias to anticipate future events also participates in the sense of continuity, thereby providing an elementary basis for the expectation of what is coming next. Husserl described the concept of a protention, which allows us to anticipate the future during the present time, an implicit, temporally defined form of which has been demonstrated experimentally by Kompass and Elliott (2001). What Husserl describes might be more easily related to conscious phenomena taking place at larger time scales, but the bias toward the latest stimulus might nonetheless be considered as an elementary mechanism subserving protention.

Related to that, it can be asked whether patients’ disrupted sense of time continuity is due to their difficulty at assigning priority to the latest occurring events. This is certainly reminiscent of the observations of Minkowski (1933), p. 259, who gave the following description: “Touché dans son dynamisme vital, le schizophrène non-seulement sent tout s’immobiliser en lui, mais est encore comme privé de l’organe nécessaire pour assimiler ce qui est dynamisme et vit au dehors,” i.e., “Not only does the patient with schizophrenia, who is affected in his vital dynamism, feel everything as coming to a halt inside him, but he also seems restricted in the very organ allowing assimilation of what is both dynamic, and exists outside.”

It might be asked how patients perceive rapid succession and so how they are able to follow a stream of incoming information over time. The impact on duration perception should also be investigated. Inasmuch duration perception relies on the accumulation of information as time moves forward, an inability to follow information over time can be expected to disturb perception duration. However, duration perception concerns time scales that are much longer than those studied here, and theoretical models relating these different time scales are missing (van Wassenhove, 2009; Grondin, 2010). More generally, the consequences of an elementary impairment at moving attention over time is necessarily speculative at this stage. Yet some possibilities seem likely in light of the known impairments described in patients with schizophrenia, and are briefly evoked in the next section.

## Possible Impact of Elementary Timing Impairments on Visual Perception

The visual environment is organized in both time and space, and several studies have shown that patients have a difficulty at organizing information in space. Our results suggest that their inability to follow and expect events over short time periods is independent of spatial impairments. It is possible that patients have specific impairments at binding information together both in space and time. However, timing difficulties might aggravate spatial difficulties in different ways.

In every day life, the outer world is usually dynamic rather than static and events succeed each other rapidly. Wind can make a tree leaves move, alternately uncovering, or occluding visual information. We usually experience no difficulty in distinguishing the tree from the objects located behind it. Yet moving objects like the moving leaves create much ambiguity in the organization of information, and some processing is required in order to attribute elements of information to the right objects. In dynamic environments, this requires discrimination in both space and time: an item has to be precisely focused in time in order to avoid confusion with distracter information, especially if it is displayed in the same location but at a different time. As a matter of fact, the use of masking experiments has shown that patients with schizophrenia have difficulties at distinguishing target information when it is closely preceded or followed by a distracter (Saccuzzo and Braff, 1981; Green and Walker, 1986; Rund, 1993; Cadenhead et al., 1998; Butler et al., 2003; Schechter et al., 2003). Explanations for this impairment are diverse (Breitmeyer, 1984; Schuck and Lee, 1989; Green et al., 1994, 2011a,b; Bedwell et al., 2003; Butler et al., 2003), but the impairment certainly confirms that patients have a difficulty with stimuli shown in close succession. Besides, based on masking experiments, it has been proposed that patients with schizophrenia have a difficulty in the temporal precision of target-directed attention (Granholm et al., 2009; Lalanne et al., 2012a). All in all, temporal difficulties mean heightened difficulties at resolving ambiguities arising in case of dynamic visual information.

Temporal impairments may also contribute to the patients’ distractibility. A Simon effect to the side of the first stimulus might indeed be similar to attentional capture, inasmuch it impedes patients to move forward in time. Such an interpretation would be consistent with a series of studies suggesting that patients are abnormally sensitive to sudden information onsets (Schwartz and Winstead, 1982; Schwartz et al., 1988; Schuck and Lee, 1989; Ducato et al., 2008), possibly related with enhanced magnocellular sensitivity (see also Laprévote et al., 2010). This hypothesis had been discarded in the literature because several studies suggested difficult detection of information known to be conveyed by the magnocellular pathway, i.e., information with a high content of low-spatial and high-temporal frequency (Butler et al., 2001, 2005; Butler and Javitt, 2005; Calderone et al., 2013, but see Skottun and Skoyles, 2007). But in fact, the results taken as a whole are reminiscent of the dissociation described above comparing explicit and implicit responses: in this case patients were excessively sensitive to the first stimulus-onset, but this effect was apparent only at an implicit level (the Simon effect) and did not help patients to make explicit judgments regarding stimuli asynchronies. This might mean that automatic response of the magnocellular pathway is increased in patients, whereas the conscious experience of the information conveyed by this pathway is impaired. The important point here is that our results would renew the hypothesis that patients are abnormally captured by information onsets, thus loosing pertinent information.

Taken together these two lines of reasoning point toward difficulties in organizing and following the flow of information. This can be expected to destabilize the patients’ representations of the outer world, and impair them in distinguishing pertinent from non-pertinent information. Further studies are required to check to which extent the basic impairments reviewed in the present work account for known difficulties in patients.

## Conclusion

Although many questions remain, our results show that information is not fused in time at very short asynchronies, either in healthy participants or in patients. Our results also indicate that healthy participants move in time very rapidly between succeeding events and that this capability is impaired in patients. These observations may mean new additional and non-conscious mechanisms underlying the sense of time continuity. It remains to be investigated whether and how these mechanisms are really involved in the sense of time continuity, and how the impairments of these mechanisms impact cognitive functions and clinical symptoms in schizophrenia.

## Conflict of Interest Statement

The authors declare that the research was conducted in the absence of any commercial or financial relationships that could be construed as a potential conflict of interest.

## Footnotes

^1^The patients with schizophrenia participating to our studies (around 20 in each study) were mildly symptomatic outpatients, usually treated with antipsychotics but without benzodiazepines. Their performance was compared with those of control participants individually matched on sex, age, and education level (Giersch et al., 2009; Lalanne et al., 2012a,b,c). We cannot firmly exclude any effect of antipsychotic medication, although dosages do not correlate with performance. In addition, it should be noted that clinical descriptions of disrupted time continuity predate the establishment of antipsychotic medication. What’s more, the mechanisms of the sense of continuous time, as well as impairments require clarification even in treated patients. All subjects gave an informed consent, which is archived by the first author.
